# Enhancing Antioxidant Retention through Varied Wall Material Combinations in Grape Spray Drying and Storage

**DOI:** 10.3390/antiox12091745

**Published:** 2023-09-10

**Authors:** Amanda Priscila Silva Nascimento, Ana Júlia de Brito Araújo Carvalho, Marcos dos Santos Lima, Samela Leal Barros, Samara Ribeiro, Matheus Pasqualli, Hugo M. Lisboa, Ana Novo Barros

**Affiliations:** 1 Post-Graduate Program Process Engineering, Federal University of Campina Grande, Av. Aprigio Veloso, 882, Campina Grande 58400-900, PB, Brazil; amandapriscil@yahoo.com.br (A.P.S.N.); samararibeiroa@gmail.com (S.R.); matheus.augusto@professor.ufcg.edu.br (M.P.); 2 Department of Food Technology, Federal Institute of Sertão Pernambucano, CEP, Petrolina 56314-522, PE, Brazil; ana.julia@ifsertao-pe.edu.br (A.J.d.B.A.C.); marcos.santos@ifsertao-pe.edu.br (M.d.S.L.); 3 Department of Food Science and Engineering, Federal University of Ceára, Av. da Universidade, 2853—Benfica, CEP, Fortaleza 60020-181, CE, Brazil; samelaleal7@gmail.com; 4 CITAB—Centre for the Research and Technology of Agro-Environmental and Biological Sciences, University of Trás-os-Montes and Alto Douro, 5000-801 Vila Real, Portugal

**Keywords:** microencapsulation, grape, phenolics, antioxidants, stability over storage

## Abstract

The encapsulation of bioactive compounds, which spans phytochemicals, vitamins, antioxidants, and other precious substances, has risen to prominence as a crucial area of interest spanning various domains, including food, pharmaceuticals, and cosmetics. This investigation delved into the efficacy of distinct wall materials—whey protein isolate, high methoxy pectin, and gum arabic—when employed individually or in combination to encapsulate and preserve phenolic compounds and antioxidants during storage. The encapsulation process involved spray-drying bioactive compounds extracted from grapes. Over a span of 120 days, the stability of these encapsulated compounds was meticulously evaluated, encompassing assessments via different antioxidant capacity assays, phenolic content analyses, and high-performance liquid chromatography measurements. The modeling of retention kinetics during storage facilitated the comprehension of the release mechanisms. Notably, the findings underscore the pivotal role of wall materials in preserving these bioactive compounds, with each material or combination of materials exhibiting varying degrees of protective capacity. Remarkably, the synergistic blend of whey protein, pectin, and gum arabic showcased the utmost retention of bioactive compounds over this study’s period. The amassed data distinctly show that an amalgamation of wall materials can indeed considerably enhance the stability of encapsulated bioactive compounds, presenting promising applications within the realms of both the food and pharmaceutical industries.

## 1. Introduction

The encapsulation of bioactive compounds, a category encompassing phytochemicals, vitamins, antioxidants, and other valuable substances, has emerged as a pivotal focal point across diverse sectors, like food, pharmaceuticals, and cosmetics [1]. Encapsulation enhances the stability, solubility, and bioavailability of these compounds, facilitating their controlled release and augmenting their sensory attributes upon integration into a final product. Among the spectrum of encapsulation methodologies, spray drying using hydrocolloids as the encapsulating materials stands out as widely favored due to its cost-effectiveness, scalability, and flexibility in accommodating a range of compounds and wall materials [2].

Carrier materials, encompassing polysaccharides or proteins that form gel-like or highly viscous dispersions in water, are commonly harnessed as encapsulation materials due to their ability to create a protective shield around the encapsulated contents. This barrier guards against environmental factors that could hasten degradation [3]. Varied hydrocolloids, due to their unique physicochemical attributes, offer distinct levels of safeguarding of the encapsulated materials, dictating their preservation and degradation patterns over time [4].

The prevalence of the microencapsulation process has been spurred by its economical operation, continuous large-scale production, high efficiency of encapsulation, extended shelf life, and protection against the thermal degradation and volatilization of the constituent materials. Protein–polysaccharide interactions play a pivotal role in the encapsulation of bioactive grape molecules. The specific nature of these interactions influences the stability, solubility, and eventual release of these compounds. This interaction is illustrated through an electrostatic attraction, wherein oppositely charged molecules are drawn together [5]. This phenomenon can lead to the formation of complex coacervates through protein and polysaccharide aggregation in a solution. This approach has successfully encapsulated grape-derived bioactives, like resveratrol and anthocyanins [6]. Tinkering with the pH or employing diverse protein and polysaccharide types can enhance the coacervate stability.

The mixture used for drying consisted of whey protein isolate, high methoxy pectin, and gum arabic, which were selected for their complementary encapsulation properties. Whey proteins, with their excellent emulsifying and film-forming properties, form a stable barrier around the phenolic compounds during spray drying [7]. High methoxy pectin, a natural carbohydrate polymer with good gelling properties, helps to encapsulate and entrap the phenolic compounds, preventing their loss during spray drying and storage. Gum arabic, a mixture of glycoproteins and polysaccharides, acts as a stabilizer and emulsifier, forming a protective barrier around the encapsulated material and enhancing its stability during spray drying and storage [8]. The combination of these three wall materials was anticipated to result in a more robust and stable encapsulation of phenolic compounds due to their complementary properties, and to enhance the overall encapsulation efficiency and retention of bioactive compounds during spray drying and storage.

Another manifestation of the protein–polysaccharide interaction is through hydrogen bonding, where molecules with polar groups are mutually attracted by sharing hydrogen atoms. This interaction is harnessed to fashion hydrogels; three-dimensional cross-linked polymer networks adept at retaining water and other molecules [9]. Hydrogels have been used to encapsulate grape-derived bioactives, like polyphenols and flavonoids [10]. The strength and resilience of these hydrogels are modulated by adjusting the cross-linking levels and polymer compositions. Lastly, another manifestation of the protein–polysaccharide interaction lies in hydrophobic interactions, where nonpolar molecules are drawn together. This phenomenon can be harnessed to create nanoparticles—minute particles capable of encapsulating grape-derived bioactive compounds, like catechins and quercetin. The size and stability of these nanoparticles can be regulated by adjusting the protein and polysaccharide types and the concentrations employed.

Despite the increasing adoption of hydrocolloids for encapsulation purposes, a significant knowledge gap exists concerning how the choice of hydrocolloids influences the retention and degradation patterns of diverse bioactive compounds over time. 

Grapes hold a substantial reservoir of polyphenols, encompassing phenolics, tannins, and flavonoids, alongside dietary fiber. As a result, they have garnered both scholarly and industrial attention due to their numerous health benefits, particularly their contributions to gut microbiota, valuable antioxidant properties, and anti-inflammatory activities [11]. Consequently, preserving these bioactive compounds becomes a fundamental endeavor. Elements, such as temperature, pH, light, oxygen, and enzymes, play a pronounced role in influencing the stability of bioactive molecules like anthocyanin. In the realm of the food industry, safeguarding these compounds presents one of the chief challenges.

Antioxidants are crucial for maintaining health as they combat oxidative stress and free radical damage. Ensuring the retention of antioxidants within encapsulated grape juice is pivotal for preserving its health-enhancing attributes. To assess the antioxidant activity, we employed three distinct assays: DPPH (2,2-diphenyl-1-picrylhydrazyl), FRAP (ferric reducing antioxidant power), and ABTS (2,2′-azino-bis(3-ethylbenzothiazoline-6-sulfonic acid)). DPPH and ABTS assays measure the ability of antioxidants to scavenge free radicals, while the FRAP assay measures the ferric-reducing ability of a sample. DPPH and ABTS typically show similar trends as they both involve the quenching of stable radicals, whereas FRAP involves the reduction of a ferric–tripyridyltriazine complex to its ferrous form [12]. Each assay targets different facets of antioxidant performance, providing a comprehensive insight into the encapsulated product’s antioxidant potential. This comprehensive approach is necessary, as different antioxidants may exhibit varying efficiencies in different assays due to their distinct mechanisms of action.

This study aims to bridge these gaps by meticulously evaluating the impact of distinct hydrocolloids—namely gum arabic, whey protein, and pectin—on the retention and degradation dynamics of a range of bioactive compounds, both immediately following encapsulation and across a 120-day period of grape juice storage. The insights derived from this investigation are poised to offer invaluable guidance for refining the encapsulation process and, ultimately, enhancing the effectiveness and applicability of encapsulated bioactive compounds across diverse fields.

## 2. Materials and Methods

### 2.1. Materials

Grape fruits of the Isabel variety were purchased at the local markets of Campina Grande City (PB, Brazil) and Petrolina City (PE, Brazil). The encapsulation agents used were Whey Protein Isolate 94% (Glanbia Provon ^®^ 292), Acacia Senegal Gum Arabica (aGpura^®^), and High Methoxy Pectin (Genu^®^).

### 2.2. Grape Juice 

The grapes were washed and sanitized in a sodium hypochlorite solution at a concentration of 50 ppm for 15 min and then rinsed in running water. Then the grapes were homogenized using a high-shear processor (Phillips Wallita, São Paulo, SP, Brazil, model RI7301, 750 W). The liquefied grapes were filtered to separate any seeds. The pulp was packed in a plastic container and stored at −4 °C until analysis. The final grape juice had a total solid content of 10.52 ± 0.03. 

### 2.3. Formulations 

In this study, four distinct formulations were employed to investigate the impact of the independent variables—comprising grape pulp concentration, gum arabic, and pectin concentration—on the dependent variables. The chosen wall materials encompassed whey (W), whey combined with pectin (W + P), whey combined with gum arabic (W + G), and whey combined with both pectin and gum arabic (W + P + G). The initial assessments established a consistent 15% whey protein isolate concentration across all formulations, while the gum arabic and pectin percentages spanned from 0 to 2%, as elaborated in Table 1. After thorough hydrocolloid solubilization, all samples underwent emulsification utilizing an IKA Ultra Turrax T-25 (IKA, Königswinter, Germany), operating at 20,000 rpm for 2 min, right before the commencement of the spray-drying experiment.

### 2.4. Spray Dryer

Spray drying (Labfirst Scientific Instruments, Shangai, China) at a PSD1 scale was used to obtain the powders. The drying conditions were defined through preliminary tests and based on the results from prior works [2,13]. An inlet/outlet temperature of 160/92 °C was used with a feed flow rate of 2.2 kg/h, and an atomization flow rate of 2.2 kg/h. 

### 2.5. Storage 

Immediately after drying, the powders were double-packed in laminated packages with a silica gel bag and duly sealed to avoid exposure to light and humidity. The powders were stored inside the mentioned packages, with outside conditions of 25 °C and 75% relative humidity for the 120 days. All characterizations were performed on the day after spray drying. Analyses of the stored materials were performed on days 30, 60, 90, and 120. 

### 2.6. Phytochemical Parameters

#### 2.6.1. Extraction and Quantification of the Individual Phenolic Compounds Using RP-HPLC/DAD

The extraction of phenolic compounds from the protein juice powder was executed utilizing the solid–liquid extraction method, following the protocol outlined by [14]. A 5 mL sample was combined with 10 mL of ethyl acetate and agitated for 5 min. The amalgamation of the resulting organic phases was followed by evaporation using a temperature-controlled rotary evaporator (IKA, Königswinter, Germany) (35 ± 1 °C). The residue from the red coupling system was then dissolved in 2 mL of 50% *v*/*v* methanol and filtered before being injected into the column.

High-performance liquid chromatography (HPLC) analyses were performed using an Agilent 1260 Infinity LC system (Agilent Technologies, Santa Clara, CA, USA), equipped with a quaternary pump (model G1311C), vacuum degasser, thermostatic column compartment (model G1316A), automatic sampler (model G1329B), diode array detector (DAD, model G1315D), and refractive index detector (RID, Model G1362A).

The compounds were separated using a Zorbax Eclipse Plus RP-C18 column (100 × 4.6 mm, 3.5 μm) and a pre-column Zorbax C18 (12.6 × 4.6 mm, 5 μm). The run time lasted 33 min. and used the following gradients: 0–5 min—5% B, 5–14 min—23% B, 14–30 min—50% B, and 30–33 min—80% B. The oven temperature was set at 35 °C and the flow rate was 0.8 mL.min^−1^ The mobile phases consisted of a 0.1 M phosphoric acid solution with pH = 2.0 (A) and methanol acidified with 0.5% phosphoric acid (B). Phenolic compounds were detected at 220 nm for (+)-catechin, (−)-epicatechin, (−)-epigallocatechin gallate, (−)-epicatechin gallate, procyanidin B1, and procyanidin B2; at 280 nm for gallic and syringic acids, hesperidin, cis-resveratrol, and naringenin; at 320 nm for caftaric acid, caffeic acid, chlorogenic acid, p-coumaric acid, and trans-resveratrol; and at 360 nm for quercetin 3- glucoside, rutin, and kaempferol [15].

Data collection and processing were performed using OpenLAB CDS ChemStation Edition (Agilent Technologies, Santa Clara, CA, USA). The external standards of the phenolic compounds were used for the calibration curves, and all the analytical curves presented R2 > 0.995. The identification and quantification of the compounds were achieved through comparison with external standards from Sigma Aldrich (St. Louis, MO, USA), including gallic acid, syngic acid, p-coumaric acid, chlorogenic acid, trans-cafaric acid, caffeic acid, hesperidin, naringenin, procyanidin B1, catechin, epicatechin, and procyanidin B2. Additional standards, including epigallocatechin, epicatechin gallate, procyanidin A2, quercetin 3-glucoside, rutin (quercetin 3-rutinoside), kaempferol 3-glycoside, and myricetin were obtained from Extrasynthesis (Genay, France). *Trans*-resveratrol and *cis*-resveratrol were sourced from the Cayman Chemical Company (Ann Arbor, MI, USA).

#### 2.6.2. Total Bioactive Content (Total Phenolics)

The total bioactive content was quantified by applying the spectrophotometric Folin–Ciocalteu method, as outlined by [16]. To initiate the process, 100 µL of residual extract, 7.90 mL of distilled water, and 0.50 mL of Folin–Ciocalteu reagent were meticulously combined within a test tube. Subsequently, after 5 min, 1.50 mL of a 20% saturated solution of Na_2_CO_3_ was introduced into the mixture, and the amalgamation was allowed to rest undisturbed for a span of 2 h. Following this, the absorbance at 765 nm was measured employing a UV-visible spectrophotometer (model UV 2000A, Instrutherm, São Paulo, SP, Brazil) within a glass cuvette possessing a 10 mm optical path. The spectrophotometer was zeroed. The resulting data, expressed in mg/kg of gallic acid equivalents, were established by means of comparison with a previously generated calibration curve. The calibration curve for gallic acid was prepared using standard solutions in the range of 0.1–50 mg/L. 

#### 2.6.3. Antioxidant Activity

The antioxidant activity was determined in triplicate using the ABTS and DPPH free radical scavenging methods, according to the methodologies described by [17,18], respectively. The Trolox analytical standard was used to construct the analytical curve and the results were expressed as Trolox equivalent per liter of extract (mmol TEAC L^−1^). 

The DPPH• (1,1-diphenyl-2-picrylhydrazyl) scavenging activity was measured spectrophotometrically at λ = 517 nm. The analysis was performed by mixing a 100 μL sample with 2.9 mL DPPH• radical ethanolic solution (100 μM), followed by incubation in the dark for 30 min. The DPPH• solution was diluted with ethanol to achieve an absorbance value of 0.950 ± 0.050 at 517 nm.

In the ABTS•+ method, the antioxidant activity was determined by the decay rate of the absorbance (λ = 754 nm) of the ABTS•+ radical, which was produced by the reaction between 5 mL of ABTS•+ 7 mM and 5 mL of potassium persulfate 2.45 mM. The mixture was kept in the dark for 16 h prior to analysis. Later, the ABTS•+ solution was diluted with ethanol to adjust the initial absorbance to 0.700 ± 0.050 at 734 nm. Then, 30 μL of the sample was added to 3.0 mL of the ABTS•+ solution and the readings were performed immediately and after 6 min of reaction.

The FRAP method was performed according to the methodology recommended by [19], with some modifications. Briefly, the FRAP reagent was prepared by mixing 25 mL of acetate buffer solution (300 mM, pH 3.6), 2.5 mL of TPTZ solution (10 mM TPTZ in 40 mM HCl), and 2.5 mL of FeCl_3_ aqueous solution (20 mM). An amount of 90 μL of the fermented beverage and 270 μL of deionized water were added to 2.7 mL of the FRAP reagent, followed by incubation at 37 °C for 30 min. Absorbance was measured at 595 nm. The results obtained were compared to a standard ferrous sulfate curve (100–2000 μmol/L).

The determination of antioxidants using FRAP was performed according to the methodology described by Rufino et al. [19]. The results obtained were compared to the standard curve of ferrous sulfate at concentrations of 100–2000 μmol L^−1^, and expressed in mmol of Fe^2+^ per liter of sample. 

### 2.7. Statistical Analysis 

All characterizations were repeated three times. The results of each characterization were assessed using a one-way ANOVA followed by Tukey’s comparisons test at 5% of probability. Software Prism 10 was used for statistical treatment and figure production.

## 3. Results

### 3.1. Effect of Wall Material Combinations on Antioxidant Activity Retention

Antioxidants are crucial for maintaining health as they combat oxidative stress and free radical damage. Ensuring the retention of antioxidants within encapsulated grape juice is pivotal for preserving its health-enhancing attributes. To assess the antioxidant activity, we employed three distinct assays: DPPH (2,2-diphenyl-1-picrylhydrazyl), FRAP (ferric reducing antioxidant power), and ABTS (2,2′-azino-bis(3-ethylbenzothiazoline-6-sulfonic acid)), each targeting different facets of antioxidant performance. This comprehensive approach offers insights into the encapsulated product’s antioxidant potential, as summarized in Table 1. 

When encapsulated with whey protein, a moderate level of antioxidant retention was observed, as indicated by the DPPH, FRAP, and ABTS values of 7.31 mg/kg, 34.99 mg/kg, and 16.23 mg/kg, respectively. This could be attributed to the whey protein creating a protective physical barrier around the active compounds, thereby maintaining a certain degree of antioxidant activity [20]. However, the efficiency of this process could be hampered by elevated drying temperatures, which may lead to protein denaturation, potentially triggering the leakage of antioxidants.

Introducing pectin alongside whey yielded divergent outcomes across the various antioxidant assays. While the DPPH value (6.55 mg/kg) showed a marginal decrease compared to whey alone, the FRAP value (41.96 mg/kg) exhibited a notable increase, indicative of a heightened ferric-reducing potential. Simultaneously, the ABTS value (14.77 mg/kg) was slightly lower than that of pure whey. The augmented FRAP value could be attributed to improved encapsulation efficiency due to the interactions between the polysaccharides and proteins. A previous study revealed that pH is a pivotal factor influencing encapsulation when utilizing whey protein concentrate and pectin [21]. The encapsulation efficiency was bolstered by forming a stronger complex between the pectin and whey protein concentrate at a pH below the isoelectric point of the latter. This interaction underscores the susceptibility of whey protein–pectin interactions to external factors, like pH, potentially influencing the encapsulated compounds’ antioxidant characteristics. The variable outcomes emphasize that the interaction between whey and pectin might differentially impact distinct types of antioxidants.

When combining whey with gum arabic, the resulting formulation exhibited the lowest DPPH value (4.42 mg/kg), indicating a comparatively diminished free radical scavenging capacity. Concurrently, the FRAP and ABTS values also dipped compared to pure whey. This decline could be attributed to the interaction between whey and gum arabic, which may not establish as robust a protective layer as the other combinations, consequently reducing antioxidant retention. Nonetheless, prior research has demonstrated that combining whey protein and gum arabic can enhance the stability and encapsulation efficiency of specific compounds, such as phenolics from grape seed extract and resveratrol [22,23]. It is important to acknowledge that the precise characteristics of the whey protein isolate and gum arabic blend can be influenced by an array of factors, including material ratio, pH, and the nature of the encapsulated compound. For instance, a previous study highlighted that a binary combination of maltodextrin and whey protein outperformed gum arabic as a wall material for squalene encapsulation [24]. 

On the other hand, the fusion of all three constituents within the wall material—whey, gum arabic, and pectin—yielded antioxidant activity comparable to other formulations, featuring a DPPH value of 6.53 mg/kg, a FRAP value of 43.68 mg/kg, and an ABTS value of 18.48 mg/kg. This suggests a potential synergistic effect, wherein the combined wall materials exert superior antioxidant protection [25]. It is evident that the composition of the wall material exerts a considerable influence on the retention of antioxidants within encapsulated grape juice. The amalgamation of whey, pectin, and gum arabic emerged as the most efficacious combination, indicating that a complex polysaccharide–protein network could enhance the safeguarding against antioxidant degradation [26]. The pH of the solution, which was an average of 4.5 in our study, plays a pivotal role in the interactions between whey, pectin, and gum arabic, and, consequently, in the encapsulation efficiency. Whey proteins, with an isoelectric point around pH 4.6–5.2, carry a net positive charge at a pH of 4.5, enabling them to form complexes with negatively charged pectin. This interaction leads to whey protein–pectin complexes, which could enhance the encapsulation efficiency by forming a stronger protective barrier around the active compounds. Conversely, the interaction between whey and gum arabic, another negatively charged polysaccharide, may not have established as robust a protective layer as the other combinations, leading to reduced antioxidant activity.

### 3.2. Effect of Wall Materials on Phenolic Compound Retention 

Phenolic compounds are a pivotal element that contribute to the antioxidant activity of grape juice [27]. Their maintenance within the encapsulated product is of paramount significance for safeguarding the health-enhancing attributes of the juice. The initial average concentrations of these compounds within the encapsulated grape juice samples immediately following the spray-drying process are thoughtfully presented in Table 2.

Starting with the whey-based encapsulation, the grape juice exhibited an initial phenolic compound concentration of 8955.14 mg/kg. Whey, as a protein-centric encapsulant, typically erects a physical barrier to counteract phenolic compound loss. However, the effectiveness of this process might be influenced by the drying phase, given proteins’ susceptibility to structural alterations under elevated temperatures, which could potentially compromise their encapsulation efficiency. Adding pectin to whey showed an enhancement in the initial phenolic compound retention, with a concentration of 10,458.75 mg/kg. Pectin, a polysaccharide, likely forges a more resilient matrix in tandem with whey, amplifying encapsulation efficiency. It could also interact with the phenolic compounds, augmenting their retention within the encapsulated structure [28].

For the grape juice encapsulated with whey and gum arabic, the phenolic compound concentration was the lowest, at 7843.99 mg/kg. Despite functioning as a collaborative encapsulating agent, the amalgamation of gum arabic and whey might not yield as robust a matrix as the whey combined with pectin. This lower concentration could also be attributed to the distinct interactions between gum arabic and phenolic compounds as compared to pectin. Similar findings have highlighted that combining whey protein with a polysaccharide improved phenolic retention [29].

In contrast, the fusion of all three hydrocolloids yielded the highest initial phenolic compound concentration of 13,741.22 mg/kg at the outset. This possibly signifies a synergistic effect, where the combined hydrocolloids establish a more robust barrier and, consequently, elevate the encapsulation efficiency and phenolic compound retention. In a nutshell, the interplay of whey, pectin, and gum arabic emerged as the most potent strategy for upholding the phenolic compounds of grape juice throughout the spray-drying process [30].

### 3.3. Effect of Wall Materials on Antioxidant Properties and Phenolic Content during Storage 

**Figure 1:**
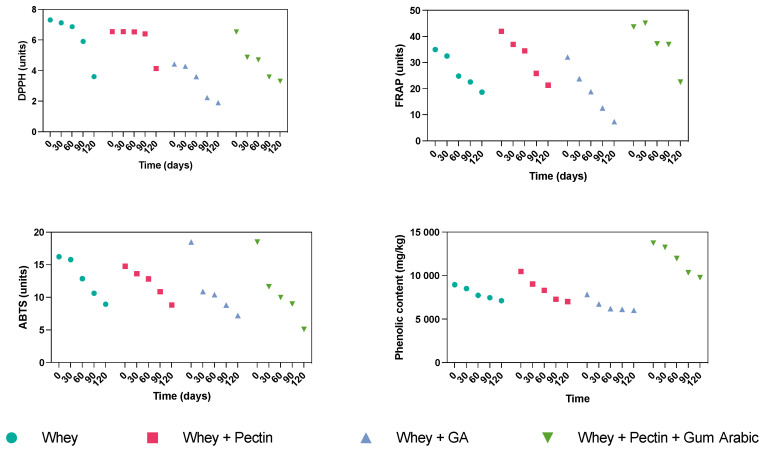
Evolution of DPPH, FRAP, ABTS, and phenolic content over the storage period of the grape juice microcapsules using different hydrocolloids as wall materials.

The investigation delved into the impact of storage duration on the retention of antioxidants and phenolic compounds within encapsulated grape juice, spanning the quartet of wall materials: whey, whey + pectin, whey + gum arabic, and whey + pectin + gum arabic. The progressive alteration of antioxidant properties throughout the 120-day storage period, assessed at four distinct time intervals, is succinctly documented in Table 2 and Figure 1. 

In the context of whey encapsulation, a declining trajectory was observed across the DPPH, FRAP, ABTS assays, and the phenolic content over the 120-day storage duration. This diminishing pattern might be ascribed to the plausible degradation and oxidation of both antioxidants and phenolic compounds over the elapsed period. Although whey proteins inherently possess protective attributes, their capacity to forestall this deterioration seems to wane as the storage duration increases. The whey + pectin encapsulation also exhibited a parallel descending pattern across all four assessment metrics throughout the storage interval. However, the pace of decline was somewhat subdued compared to whey alone; this was particularly evident in the cases of FRAP and phenolic content. This underscores that incorporating pectin might bolster the stability of antioxidants and phenolic compounds within encapsulated grape juice, which is potentially attributable to the heightened structural integrity of the encapsulating matrix. Pectin’s effectiveness in enhancing the stability of bioactive compounds has been acknowledged in diverse encapsulation studies. For instance, low methoxyl pectin-coated liposomes amplified resveratrol and epigallocatechin gallate stability in orange juice [31].

Conversely, the whey + gum arabic encapsulation displayed a significant decrease across all the evaluated metrics, marked by pronounced DPPH, FRAP, and phenolic content drops. In contrast, the ABTS values gradually declined, implying that this wall material might better safeguard certain antioxidants.

In conclusion, the synergistic amalgamation of whey, pectin, and gum arabic showcased the most efficacious encapsulation performance over the storage period. Despite the overall decline in DPPH, FRAP, and ABTS values, the phenolic content followed a distinct trajectory. It initially increased until the 90-day mark, followed by a slight dip at the 120-day point, yet consistently maintained the highest level among all the wall materials. This distinctive behavior could potentially stem from the cumulative effect of the three components, yielding heightened protection for the phenolic compounds against degradation. It is well-recognized that the duration of storage profoundly influences the retention of antioxidants and phenolic compounds [32]. While all the wall materials showed a gradual decline in retention over the 120 days, the amalgamation of whey, pectin, and gum arabic exhibited superior preservation characteristics, particularly concerning the phenolic compounds.

### 3.4. Effect of Wall Materials on the Kinetics of Degradation of the Phenolic Compounds

Table 3 presents the results for modeling the degradation kinetics of the different encapsulation materials. Whey alone, as an encapsulation wall material, shows a gradual reduction in all antioxidant parameters throughout the storage duration. Modeled using first-order kinetics, this decline follows a relatively consistent trajectory, signifying a persistent antioxidant loss. Nonetheless, whey imparts a moderate degree of safeguarding, particularly during the initial storage phase.

When combined with pectin, whey introduces a slightly elevated level of protection for most antioxidants, as compared to whey used in isolation. This effect is especially prominent during the initial storage period. However, the pace of antioxidant diminishment escalates over time, as demonstrated by the higher rate constant in the first-order model. This observation implies that while pectin can enhance initial antioxidant stability, its impact on long-term retention might be limited.

When whey is harmonized with gum arabic, a substantial enhancement in antioxidant retention emerges, particularly in the context of phenolic compounds and FRAP. Similar findings were previously reported for the encapsulation of flavonoids from citrus fruits [33]. Despite the higher rate constant in the first-order model, signifying an accelerated antioxidant loss rate, the initial concentrations of these antioxidants substantially exceed those in isolation, hinting at an improved initial encapsulation or protection offered by gum arabic.

Combining all three materials showcases the most commendable overall performance concerning antioxidant retention. Despite a relatively elevated loss rate, as indicated by the first-order model, the initial antioxidant concentrations significantly surpassed those found using the other wall materials, underscoring the superior level of safeguarding. This enhancement could potentially be attributed to the synergistic effects of pectin and gum arabic when partnered with whey, which bolsters encapsulation efficiency and antioxidant stability.

The kinetic data for the phenolic compounds, DPPH, FRAP, and ABTS retention were rigorously modeled using zero-order and first-order kinetics, and spanned the various encapsulation wall materials. The zero-order kinetic model assumed a steady reaction rate regardless of the reacting species concentration. The initial concentrations (B0) of phenolic compounds, DPPH, FRAP, and ABTS are maximized in the whey + pectin + gum arabic encapsulations, harmonizing with prior experimental outcomes. The rate of decline (B1) indicates a swifter antioxidant loss with certain wall materials, notably the W + P and W + P + G encapsulations for the phenolic compounds, W + G and W + P + G for DPPH, W + G for FRAP, and W + P + G for ABTS. This suggests a more accelerated antioxidant degradation or release from these encapsulations. The goodness-of-fit (R^2^) for the zero-order model generally exhibits strong results across all the wall materials and antioxidants. However, some of the fits could be further refined, particularly that of W + G for the phenolic compounds and W + P for DPPH.

In the context of the first-order model, which presumes that the reaction rate is proportionate to the concentration of reacting species [34], the initial concentrations (Y0) closely mirror those of the zero-order model. The first-order model’s rate constants (K) exhibit more variation across the wall materials and antioxidants. The highest K value for the phenolic compounds is within the W + G encapsulation, signifying a more rapid degradation rate. Comparable trends exist for DPPH with W + P + G, while for FRAP and ABTS, the highest rate constants manifest with W + G and W + P + G, respectively, again indicating expedited degradation with these wall materials. The R-squared values for the first-order model match or outperform the zero-order model for most of the combinations, with notably robust fits for the W + P and W + G encapsulations across all the antioxidants. Taken together, the kinetic modeling substantiates the conclusion that the combination of whey, pectin, and gum arabic within the encapsulation wall may decelerate the phenolic compounds’ degradation and sustain antioxidant activity over time.

The synergistic outcome of synergizing whey protein, pectin, and gum arabic as encapsulation materials can be attributed to their unique structural and functional attributes. Renowned for crafting a gel-like network, whey protein effectively immobilizes phenolic compounds [35]. Pectin, renowned for its adeptness in film formation and water binding, likely introduces an extra protective layer, thereby extending antioxidant retention [36,37]. Gum arabic, with its emulsifying prowess, bolsters the stability of the encapsulated phenolic compounds, thereby ameliorating retention efficiency [33]. 

Merging these three hydrocolloids likely creates a multifaceted, structurally intricate matrix that confers heightened protection to the phenolic compounds. This notion finds support in the fact that gum arabic and whey protein concentrate, when combined, have been proven to yield elevated encapsulation efficiency and a commendable retention of volatiles [38].

The synergistic interplay between whey protein, pectin, and gum arabic as encapsulation materials can be attributed to their distinct yet complementary structural and functional attributes. This fusion yields a multi-tiered, structurally intricate matrix that furnishes a superior safeguarding of phenolic compounds and other antioxidants, presenting a promising avenue for applications in various sectors. The encapsulation material’s suitability for storage use, as presented in Table 3, indicates that the combination of whey, pectin, and gum arabic has the most commendable overall performance concerning antioxidant retention, despite a relatively elevated loss rate indicated by the first-order model. This superior level of safeguarding is attributed to the synergistic effects of pectin and gum arabic when partnered with whey, which bolsters encapsulation efficiency and antioxidant stability. However, the kinetic modeling of the phenolic compounds, DPPH, FRAP, and ABTS retention using zero-order and first-order kinetics reveals that antioxidants still degrade over time. The rate of decline (B1 or K) indicates a swifter antioxidant loss with certain wall materials, notably the W + P and W + P + G encapsulations for phenolic compounds, W + G and W + P + G for DPPH, W + G for FRAP, and W + P + G for ABTS. This suggests a more accelerated antioxidant degradation or release from these encapsulations.

The degradation of the encapsulation materials, whey protein, pectin, and gum arabic, can be correlated with the assumptions made for the zero- and first-order kinetics. The zero-order kinetic model assumes a steady reaction rate regardless of the reacting species concentration, while the first-order model presumes that the reaction rate is proportionate to the concentration of reacting species. The structure of the encapsulation materials can degrade over time due to oxidation, Maillard browning, and hydrolysis, leading to a reduction in molecular weight and a change in the physical and chemical properties of the encapsulation material. This degradation of the encapsulation material can affect the encapsulation efficiency and lead to the release of the encapsulated antioxidants, as indicated by the kinetic modeling. Therefore, the structural degradation of the encapsulation materials is correlated with the zero- and first-order kinetic degradation of the antioxidants, as the storage conditions and the natural degradation processes of the materials influence both.

### 3.5. Effect of Wall Materials on Specific Phenolic and Antioxidant Molecules Retention after Spray Drying 

**Figure 2:**
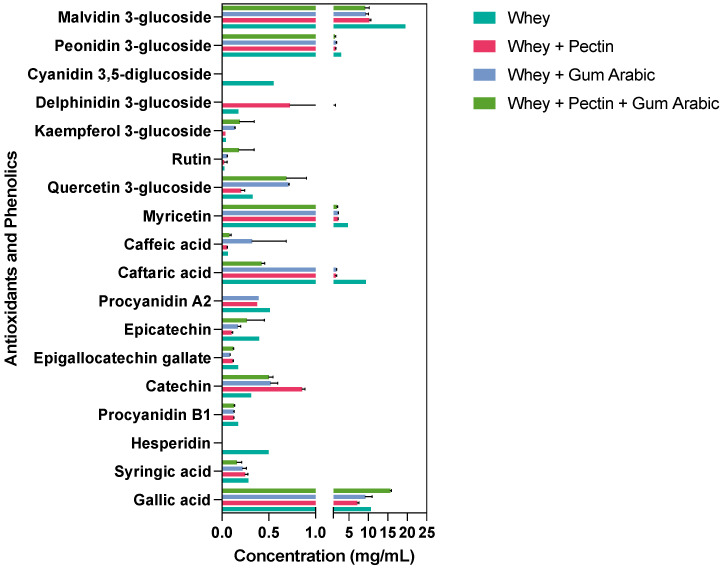
Retention of antioxidants and phenolics using different wall materials immediately after spray drying.

We meticulously investigated the impact of diverse wall materials on the stability of phenolic compounds post-spray-drying encapsulation. Our findings unveil distinctive trends that underscore the pivotal role of judicious wall material selection in optimizing phenolic compounds’ stability and potential bioavailability. Figure 2 presents an overview of these compounds’ retention immediately after spray drying. Notably, our results showcase gallic acid’s highest content when encapsulated in synergy with whey, pectin, and gum arabic. This intriguing observation suggests that a synergistic blend of wall materials could foster the enhanced preservation of gallic acid compared to standalone choices. Conversely, syringic acid exhibited heightened stability when coupled with whey alone and the whey + gum arabic amalgamation, suggesting a nuanced interplay between wall materials and specific phenolic compounds’ stability.

Intriguingly, hesperidin, a noteworthy phenolic compound, was solely detected in the context of the whey wall material treatment, implying its potential sensitivity to conditions introduced by other wall material components. The robust stability of procyanidin B1, catechin, and epigallocatechin gallate across all the encapsulation treatments underscore their resilience. Epicatechin, however, demonstrated an elevated content in both the whey and whey + pectin + gum arabic treatments, emphasizing that certain compounds might exhibit improved stability in response to a combination of wall materials. A captivating facet emerged with caftaric acid, which experienced a significant reduction upon encapsulation with the combined wall materials, particularly the whey + pectin + gum arabic blend. In contrast, caffeic acid responded well to all the treatments except to whey alone, signifying its enhanced stability within the ambit of combined wall materials.

Interestingly, our assessment of other phenolic compounds, including myricetin, quercetin 3-glucoside, rutin, and kaempferol 3-glucoside, highlighted a diversity of outcomes, which were contingent on the chosen wall material. Delphinidin 3-glucoside exclusively emerged within the whey + gum arabic treatment, suggesting a tailored safeguarding effect attributed to this specific combination. Anthocyanins, exemplified by peonidin 3-glucoside and malvidin 3-glucoside, were found in all the treatments, with varying content levels, reaching their zenith in whey alone and their nadir in whey + pectin + gum arabic.

Our exploration revealed hesperidin’s exclusive presence within the whey treatment, hinting at potential interactions with whey proteins that augment its stability or encapsulation efficiency [39]. This could emanate from hydrophobic interactions or hydrogen bonding, culminating in a robust matrix that is able to withstand the rigors of spray drying. The augmented stability of syringic acid, notably in the whey alone and whey + gum arabic treatments, potentially is owing to gum arabic’s intricate structure involving proteinaceous and sugar components, which might form specific bonds with syringic acid, ensuring stability through the spray-drying process [40]. The pronounced affinity of epicatechin towards the whey and whey + pectin + gum arabic treatments underscores its preferential interactions with these wall materials, potentially driven by the interplay of the hydroxyl groups inherent in epicatechin with the polysaccharide or protein constituents of these chosen wall materials [41].

Nonetheless, these interactions between phenolic compounds and wall materials likely encompass a medley of bonds, including hydrogen bonds and hydrophobic interactions. Their intricate nature is inevitably influenced by factors such as pH, temperature, and ionic strength during encapsulation. A deeper comprehension of these interactions at a molecular level necessitates targeted investigations encompassing spectroscopic analysis, molecular modeling, and other advanced techniques.

### 3.6. Effect of Different Hydrocolloids on the Stability of Antioxidant and Phenolic Compounds during the Storage Period

**Figure 3:**
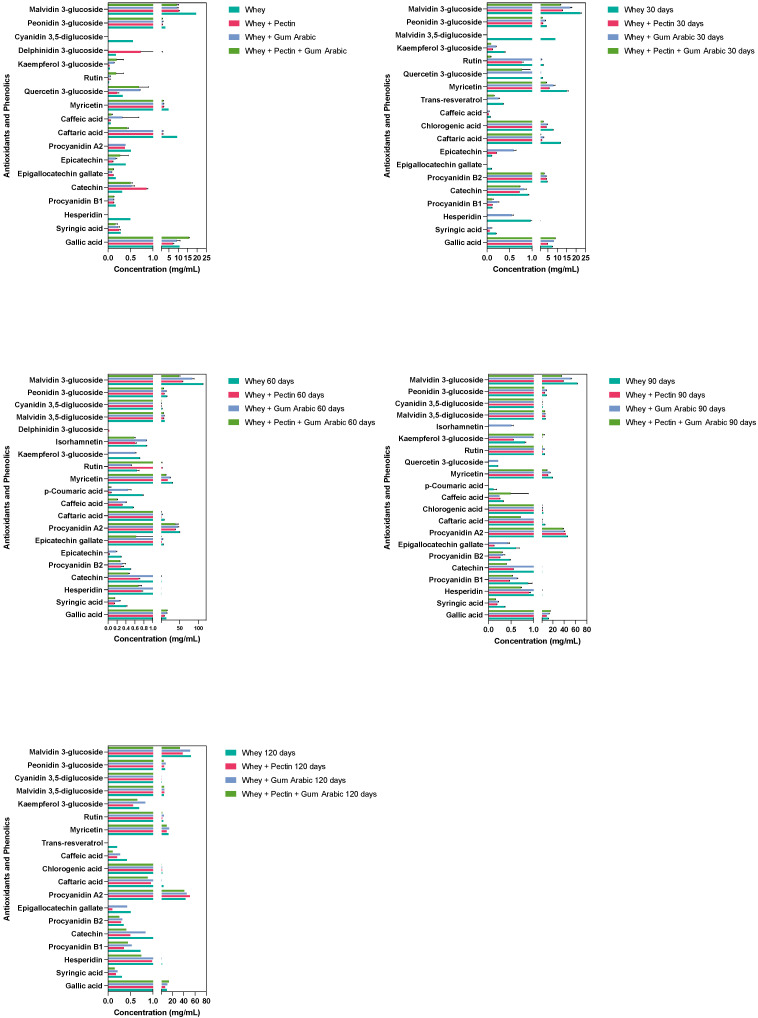
Evolution of the antioxidant phenolic contents of the grape juice microcapsules using different hydrocolloids as wall materials over the storage period.

The microencapsulation process was harnessed, employing a diverse array of wall materials—whey, pectin, gum arabic, and their hybrid combinations—to ascertain their efficacy in safeguarding an assortment of bioactive compounds across a 120-day timeline. These compounds’ stability within the microcapsules was monitored via high-performance liquid chromatography (HPLC), which furnished profound insights into how the encapsulating materials influenced the degradation kinetics of these pivotal bioactive entities. A visual representation of the compound concentrations recorded over the course of the 120 days is illustrated in Figure 3.

Spanning the observed formulations, gallic acid concentrations consistently declined throughout the 120-day interval. However, the most notable retention occurred in the microcapsules fashioned from the amalgamation of pectin, gum arabic, and whey. This intriguing observation possibly signifies a synergistic alliance between these materials, which potentiate to shield gallic acid against deterioration. Hesperidin, which retained its stability solely within the whey-based formulation, remained undetectable in the other tested materials. This disparity might hint at the intricate interactions between hesperidin, pectin, and/or gum arabic, culminating in degradation, complexation, or binding that renders it elusive for detection.

Procyanidin A2, in stark contrast, exhibited an upsurge across all the formulations, with the formulation encompassing whey, gum arabic, and pectin demonstrating the least variation over this study’s duration. This outcome underscores the potential protective envelope conferred by this combination, potentially curbing procyanidin A2’s degradation or thwarting its transformation into other compounds. Catechin concentrations ebbed across all the formulations over this study’s trajectory. Yet, the decline was less pronounced in the whey-only and whey + pectin formulations, suggesting these configurations could offer enhanced catechin stability.

Strikingly, the myricetin concentration showed a tendency to escalate across all the formulations as time elapsed. Notably, the whey and gum arabic formulation exhibited the most modest increase, hinting at a decelerated degradation or transformation rate vis-à-vis the other compounds. Peonidin 3-glucoside and malvidin 3-glucoside mirrored similar patterns, with concentrations showing initial increments for 60 days, followed by a dip in the former and a sustained surge in the latter. The whey + gum arabic + pectin formulation charted the most gradual degradation trajectory, implying heightened compound stability within this composite synergy.

The refinement of emulsification processes before spray drying could conceivably bolster the encapsulation efficiency and fortify the stability of the bioactive compounds ensconced within hydrocolloids. Elevated emulsification accords a uniform dispersion of bioactive entities within a hydrocolloid solution, culminating in more efficacious encapsulation during spray drying. Consequently, this might yield an augmented retention of these compounds at genesis (time zero), amplifying their fortification against degradation over protracted periods—in this instance, the stipulated 120 days.

Moreover, emulsification’s salutary effects extend to microcapsule size reduction post-spray drying. This yields microcapsules with a higher surface-area-to-volume ratio, engendering a sturdier partition between the encapsulated compound and extraneous environmental influences, ergo hampering the degradation trajectory. Additionally, emulsification precludes the peril of droplet coalescence, mitigating the prospect of bioactive compound loss or deterioration during the drying process.

Considering the diverse hydrocolloids employed as wall materials in this inquiry, variations in their molecular architecture and composition potentially usher in differential encapsulation efficiencies and safeguarding mechanisms for the compounds in question. Hydrocolloids boasting intricate molecular frameworks, such as gum arabic, could ostensibly furnish a more resilient physical barricade against degradation. Nevertheless, myriad factors—encompassing the interplay between a hydrocolloid and a specific bioactive compound, environmental parameters, and the hydrocolloid’s inherent properties—might also interact to influence the degradation kinetics.

However, the potential dividends of enhanced emulsification should be measured against the backdrop of various factors, including the precise attributes of the hydrocolloid and bioactive compound, the chosen emulsification technique, and the spray-drying conditions. Subsequently, undertaking further empirical investigations would be a prudent trajectory, unraveling the precise extent to which heightened emulsification can optimize compound retention and bolster stability within this dynamic system.

## 4. Conclusions

In summary, this investigation serves to highlight the pivotal role that different wall materials play in the encapsulation and preservation of phenolic compounds and antioxidants throughout extended storage periods. Of particular significance is the remarkable efficacy showcased by the synergistic combination of whey protein isolate, high methoxy pectin, and gum arabic. This combination demonstrates an impressive ability to collaboratively enhance the protection and retention of these crucial bioactive constituents, suggesting a potential cooperative mechanism at play.

These findings not only advance our understanding of the encapsulation process, but also shed light on its profound influence on the long-term stability of bioactive compounds. As a result, new avenues are unveiled for the optimization of microencapsulation techniques in both the expansive food industry and the dynamic realm of pharmaceuticals. The insights gleaned from this study may prompt researchers and practitioners to explore innovative strategies for fortifying the encapsulation process, thereby augmenting the shelf life and bioavailability of encapsulated bioactive compounds.

To propel these discoveries further, it is imperative that subsequent research delves into the intricacies of the mechanisms underpinning the observed outcomes. Investigating the molecular interactions between wall materials and bioactive compounds, as well as delving into the influence of processing conditions, pH variations, and other environmental factors, could provide a deeper understanding of the phenomenon. This holistic exploration will enable us to tailor microencapsulation strategies more precisely, optimizing the proportions of wall materials to achieve the maximal retention and stability of these invaluable bioactive compounds.

## Figures and Tables

**Table 1:** Grape juice formulations encapsulated using a spray dryer with whey protein, gum arabic, and pectin.

Experiments	Units	Grape Pulp	Whey Protein	Pectin	Gum Arabic
W	g/100 g	85%	15%	-	-
P	g/100 g	83%	15%	2%	-
G	g/100 g	83%	15%	-	2%
M	g/100 g	81%	15%	2%	2%
pH		4.38 ± 0.012 ^b^	4.41 ± 0.08 ^b^	4.47 ± 0.06 ^a^	4.46 ± 0.01 ^ab^
Spray-drying conditions					
Drying gas flow rate	Kg/h	200 ± 5.0	200 ± 5.0	200 ± 5.0	200 ± 5.0
Feed flow rate	kg/h	2.2 ± 0.1	2.2 ± 0.1	2.2 ± 0.1	2.2 ± 0.1
Atomization flow rate	Kg/h	2.2 ± 0.2	2.2 ± 0.2	2.2 ± 0.2	2.2 ± 0.2
Inlet temperature	°C	160 ± 2	160 ± 2	160 ± 2	160 ± 2
Outlet temperature	°C	91 ± 1	91 ± 1	91 ± 1	91 ± 1

Note: Different letters indicate statistically significant differences (*p* < 0.05). W—formulation with whey; P—pectin formulation with whey; G—gum arabic formulation with whey; M—Mixed formulation with a mixture of whey, HM pectin, and gum arabic.

**Table 2:** Results of antioxidant activity using DPPH, ABTS^+^, FT, and FRAP methods on grape juice stored for 120 days.

Time	Method	Experiments			
		Whey	Whey + Pectin	Whey + Gum Arabic	Whey + Pectin + Gum Arabic
0 days	DPPH (mmol TEAC L^−1^)	7.31 ± 0.12 ^a^	6.53 ± 0.12 ^abc^	4.42 ± 0.55 ^def^	6.53 ± 0.25 ^abc^
	FRAP	34.98 ± 0.14 ^bc^	41 ± 1.34 ^a^	43.68 ± 0.02 ^a^	45.16 ± 0.18 ^a^
	ABTS	16.22 ± 0.21 ^a^	14.77 ± 0.63 ^ab^	14 ± 3.39 ^ab^	12.09 ± 0.51 ^bcde^
	Phenolic content	8955 ± 21.37 ^cdefg^	10,458 ± 245 ^bc^	7843 ± 91.55 ^fghi^	9770 ± 74.91 ^cde^
30 days	DPPH (mmol TEAC L^−1^)	6.87 ± 0.65 ^ab^	6.39 ± 0.06 ^abcd^	4.27 ± 0.30 ^efg^	4.86 ± 0.08 ^bcdef^
	FRAP	32.47 ± 0.37 ^cd^	35 ± 1.71 ^bc^	29 ± 1.29 ^d^	43.68 ± 0.02 ^a^
	ABTS	15.79 ± 0.14 ^a^	13.61 ± 0.64 ^abc^	10.89 ± 0.33 ^cde^	11.64 ± 0.53 ^bcde^
	Phenolic content	8511 ± 111.37 ^defgh^	9031 ± 29.13 ^cdef^	6116 ± 212.24 ^i^	10,326 ± 29.13 ^bcd^
60 days	DPPH (mmol TEAC L^−1^)	6.61 ± 0.08 ^abc^	6.54 ± 0.62 ^abc^	3.60 ± 0.03 ^fgh^	4.71 ± 0.65 ^cdef^
	FRAP	24.82 ± 0.02 ^ef^	34.48 ± 0.05 ^bc^	23.75 ± 0.91 ^efg^	36.95 ± 0.41 ^b^
	ABTS	14 ± 1.98 ^ab^	12.83 ± 0.24 ^abcd^	10.40 ± 0.34 ^cdef^	9.99 ± 0.05 ^cdef^
	Phenolic content	7461 ± 103.98 ^fghi^	8307 ± 66.58 ^efgh^	6207 ± 82.24	11,972 ± 29.13 ^ab^
90 days	DPPH (mmol TEAC L^−1^)	5.91 ± 0.12 ^abcde^	5.98 ± 0.93 ^abcde^	2.23 ± 0.67 ^gh^	3.58 ± 0.05 ^fgh^
	FRAP	22.01 ± 0.91 ^fgh^	20 ± 1.05 ^gh^	18.82 ± 0.62 ^h^	33 ± 2.93 ^bcd^
	ABTS	10.62 ± 0.19 ^cdef^	10.87 ± 0.05 ^cde^	8.80 ± 0.75 ^ef^	8.98 ± 0.25 ^ef^
	Phenolic content	7736 ± 87.15 ^fghi^	7023 ± 253.22 ^hi^	6003.97 ± 187.2	13,741 ± 240.1 ^a^
120 days	DPPH (mmol TEAC L^−1^)	3.60 ± 0.01 ^fgh^	4.13 ± 0.03 ^efg^	2.11 ± 0.23 ^h^	3.31 ± 0.78 ^fgh^
	FRAP	18.63 ± 0.42 ^h^	25.87 ± 0.51 ^e^	12.57 ± 0.44 ^i^	21.69 ± 0.79 ^fgh^
	ABTS	8.93 ± 0.08 ^ef^	9.21 ± 0.33 ^def^	7.21 ± 0.05 ^fg^	5.11 ± 0.08 ^g^
	Phenolic content	7122 ± 14.56 ^ghi^	7293 ± 66.58 ^fgh^	6732 ± 44.94 ^hi^	13,251 ± 362 ^a^

Note: The means followed by the same letter in the columns and lines do not differ statistically using Tukey’s test at 5% probability. Results are expressed as the mean ± standard deviation (n = 3). W—formulation only with whey; P—formulation with whey and pectin; G—formulation with whey and gum arabic; M—formulation with the mixture of encapsulants.

**Table 3:** Kinetic modeling of antioxidant and phenolic compounds loss over storage period.

	Whey	Whey + Pectin	Whey + Gum Arabic	Whey + Pectin + Gum Arabic
Zero-order kinetic model—Phenolic content				
B0	8901	10,145	7440	13,986
B1	−15.72	−28.7	−14.32	−36.22
R2	0.9691	0.9547	0.8004	0.9683
First-order kinetic model—Phenolic content				
Y0	8998	10,448	7848	13,986
K	−0.006664	−0.0112	−0.03096	−0.00000441
R squared	0.9837	0.992	0.9981	0.9683
Zero-order kinetic model—DPPH				
B0	7.888	7.028	4.702	6.146
B1	−0.02873	−0.01661	−0.02357	−0.02573
R squared	0.7955	0.5486	0.9293	0.9169
First-order kinetic model—DPPH				
Y0	7.888	7.028	4.702	6.449
K	−0.000003144	−0.000003401	−0.000006607	−0.01271
R squared	0.7954	0.5486	0.9292	0.957
Zero-order kinetic model—FRAP				
B0	35.22	42.59	31.04	47.21
B1	−0.1421	−0.1745	−0.2022	−0.1685
R squared	0.9676	0.9741	0.9923	0.7955
First-order kinetic model—FRAP				
Y0	35.56	42.59	31.76	47.21
K	−0.002602	−0.000001832	−0.003903	−3.795 × 10^−6^
R squared	0.9699	0.9741	0.9968	0.7954
Zero-order kinetic model—ABTS				
B0	16.84	15.11	16.08	16.72
B1	−0.06582	−0.04878	−0.08206	−0.09801
R squared	0.9669	0.966	0.8044	0.9003
First-order kinetic model—ABTS				
Y0	16.84	15.11	18.34	18.09
K	−0.000002279	−0.000001034	−0.03353	−0.01551
R squared	0.9669	0.966	0.9622	0.9457

## Data Availability

Data is contained within the article.
